# How to safely and smoothly resume trials after the COVID-19 outbreak

**DOI:** 10.7189/jogh.10.020310

**Published:** 2020-12

**Authors:** Zejuan Wang, Gang Chen, Xiaona Liu, Aihua Du, Min Li, Jin Wang

**Affiliations:** Department of Clinical Pharmacology, Aerospace Center Hospital, Peking University Aerospace School of Clinical Medicine, Beijing, China

Due to the COVID-19 outbreak, routine clinical trial initiations and enrollments (except those for COVID-19) announced by many hospital institutions were suspended. Since mid-March, under the epidemic prevention and control efforts in China, many enterprises have resumed work and production in an orderly manner [1]. In a policy issued March 21, the Beijing Municipal Health Commission encouraged the resumption of daily medical services for all hospitals, while strengthening epidemic control work and announcing that patients with signs and symptoms of COVID-19 visit doctors in three specific hospitals [2].

Unlike the patient, most research subjects (including healthy volunteers) are volunteers and mostly have other options. However, besides investigators, research subjects involved in drug research and development also play an essential role in resuming trials, especially those for COVID-19. From an ethical perspective, the safety and well-being of human subjects are always a priority over science [3,4]. This epidemic is a long-term, dynamic event that will require nearly constant, proactive strategy development and problem solving during the global outbreak. Asymptomatic carriers of the virus may be among patients, health care workers, or visitors in hospitals. Unlike the patients, who are usually managed individually in trials, the number of healthy subjects enrolled in phase I trials ranges from 8 to 30+ in each group. Therefore, protecting the subjects, including healthy volunteers, during clinical trial resumption should be considered by investigators and the Institutional Review Board(IRB).Since March, Our center’s experience (**Figure 1**) in each step and process may be a reference for other research centers in this situation.

**Figure 1:**
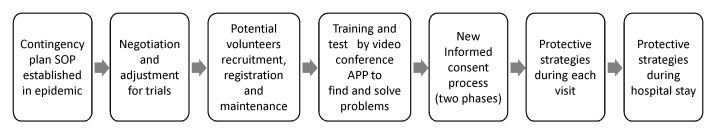
The strategies of trials resumption in each process.

## STRATEGIES

First and foremost, to protect the subjects, Standard Operating Procedures (SOPs) regarding the operation management and quality control of clinical trials during the epidemic should be established to instruct the daily preventive controls and the contingency responses to different situations, according to the Good Clinical Practice(GCP), the Declaration of Helsinki, and the applicable regulatory requirements and guidance (including those for COVID-19 prevention). Regarding the informed process, giving a detailed explanation using a cloud video meeting app on any mobile phone, ipad or computer before signing and dating paper-based or electronic informed consent forms (ICFs) can reduce the frequency and length of visits, as well as close contact with others. The daily preventive control measures for individuals include recording epidemiological history, routine protection with masks and hand washing, daily temperature tracking, and reporting signs and symptoms, which are required for all persons (including staff and subjects) visiting our department. Environment management is another fundamental measure to strengthen during nosocomial infection control.

**Figure:**
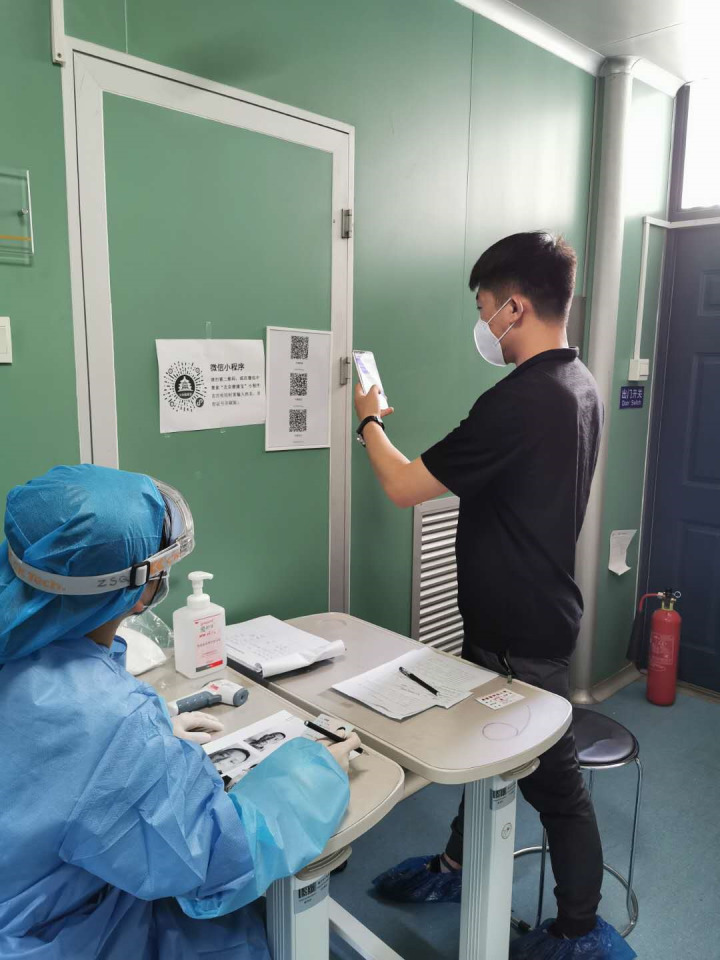
Photo: Research staff wearing preventive kits are identifying the subjects with ID Card and videoconferencing photo in the buffer zone at site after hand-washing with antibacterial gel and initial assessment of COVID-19 with questionnaires and subjects are scanning the two-dimensional code for showing itinerary information (from the collection of Dr Jin Wang, used with permission).

Second, a timely negotiation with the sponsor and the IRB about the contract, protocols, schedule, process, data collection, drug and sample management, urgent changes and reports by risk evaluation and mitigation strategies is important to protect subjects and minimize impacts on data integrity in different situations. As local regulations demand that all persons should cooperate with some screening and monitoring procedures for COVID-19 when visiting the hospital, the ICF and recruitment advertisement should be amended to illustrate that “participants are informed and required to comply with the related non-trial measures during these special circumstances.” However, protocol amendment maybe not needed if the data collected are not part of a new research objective. These would then be reviewed and approved by the IRB. The exact measures required by the regulations at that time should be quickly submitted to the IRB as supplementary materials before subjects participate in trials, and continuous informed consent is necessary.

Third, potential volunteers are recruited and retained online by specific recruitment and medical staff in advance through social media networking channels like Facebook, LinkedIn, Twitter, QQ, WeChat etc. Epidemiological history, such as itinerary, close contacts, physical condition, and certain credentials over the last 14 days or more are registered and tracked until the last trial visit. Specific recruitment staff explain the possible delay in start time, how participants can protect themselves in their daily lives, and how to use a video meeting application.

Fourth, before trial initiation, all investigators participate in a video conference to learn about SOPs, regulations, and the informed consent process to identify and improve any problems that could continuously arise. Investigators should develop and prepare materials for informed consent regarding the trial, protections, and exact screening and monitoring measures for COVID-19.

Fifth, the informed consent process is divided into two phases: a cloud video meeting first and then face-to-face discussions between the investigator and each subject. In the first online period, at roll call, a photo of each subject is taken with permission for later on-site in person visitor identification. Then, the ICF, the trial schedule, the regulations participants are required to comply with, the seven-step hand washing method, how to protect oneself at home and while traveling, the special procedure demonstration and environment introduction, etc., are explained in detail. Meanwhile, the subjects are informed of the possibility of synchronous audio and video recording and reminded by staff if they are offline or not in front of the camera. Finally, the investigator answers any subjects’ questions, provides the contact information, and announces the next face-to-face discussions. Before one-on-one discussions on-site, the volunteers are surveyed the satisfaction with the video meeting and informed status by questionnaires. Then the investigator discusses informed consent with each one, interacting more with the subjects offline, out of the camera, or those who were not satisfied.

Sixth, based on the new process and strategies for each on-site visit of subjects, the environment zones are set and divided into a clean zone, buffer zone, and contaminated zone by investigators. The investigator should make an appointment with each subject individually to avoid close group contact as before. Measures taken during each on-site visit include mask wearing and hand washing, temperature measurements, one-meter distance at least between each one, itinerary verification, ID checks using the photo obtained during the initial video call, and epidemiological history as required by regulation requirements. Protective face and eye masks and gloves are required for the investigators, especially during oral examination.

Seventh, the isolation ward is prepared for the subjects before and during hospital stay during the COVID-19 contingency. An environment with suitable temperature and humidity, good air circulation, and object surface disinfection are key points to keep subjects under good conditions when they stay in the hospital. The bed space between each subject with a mask is maximized or separate room could be assigned if feasible. It is better to stagger the time to eat if protocol is allowed.

## IMPLICATIONS AND CONCERNS

The COVID-19 epidemic will pass, and all trials will be restored gradually. Research and development of drugs or vaccines will continue for public health and well-being. During trial resumption, the seven steps described above may be effective in reducing the anxieties of research subjects and staff, and protect them from the virus. The practice has shown that, in the COVID-19 era, remote videoconferencing is an optional tool to reduce the chance of close contact, crowdedness, and possible risk of infection in trial resumption, and has worked effectively in our clinical trials. Though it may be due to reductions in time, cost, and anxiety of transmission, subjects support this digital approach with high satisfaction. From an ethical perspective, key technology concerns include their data protection, privacy, confidentiality and representativeness of human subjects, appropriate authorizations, identification, and quality of informed consent [5]. The potential value of remote videoconferencing, remote monitoring and other digital technologies during and after trial resumption may be further investigated to increase productivity and reduce the cost of conducting a trial in the future.
